# Case Report: Arthroscopic minimally invasive treatment of pediatric calcaneal fracture

**DOI:** 10.3389/fped.2026.1838911

**Published:** 2026-06-11

**Authors:** Li Zhiye, Wang Ye, Ren Zhaohui, Lv Guangren

**Affiliations:** Bone and Joint Rehabilitation Center, Xiangya Boai Rehabilitation Hospital, Changsha, Hunan Province, China

**Keywords:** internal fixation, minimally invasive surgery, pediatric calcaneal fracture, percutaneous leverage reduction, subtalar arthroscopy

## Abstract

**Background:**

Pediatric calcaneal fractures are rare and often result from high-energy trauma. Traditional open reduction carries risks of wound complications and soft tissue damage. We report a case of a 13-year-old girl with a comminuted intra-articular calcaneal fracture treated successfully with subtalar arthroscopy-assisted percutaneous reduction and internal fixation.

**Case summary:**

A 13-year-old girl presented with left heel pain after a fall from a height. Imaging revealed a comminuted calcaneal fracture with posterior facet depression. Under general anesthesia, subtalar arthroscopy was used to visualize and reduce the depressed posterior facet percutaneously, followed by fixation with multiple Kirschner wires. The patient achieved fracture union by 2 months and regained full function with an AOFAS score of 95 at 3 months. No complications occurred.

**Conclusion:**

Subtalar arthroscopy-assisted percutaneous fixation is a safe and effective minimally invasive option for selected pediatric calcaneal fractures, offering excellent visualization, anatomical reduction, and rapid recovery.

## Introduction

The calcaneus is the most commonly fractured tarsal bone, accounting for about 60% of all tarsal fractures. Calcaneal fractures are very common in adults but rare in children (1, 2). In this case, the patient had a fracture with severe posterior facet depression. Since conservative treatment couldn't restore the articular surface and open reduction posed risks like trauma and infection, an arthroscopically-assisted reduction was chosen. This minimally invasive approach allows joint debridement and reduces the risk of subtalar arthritis, leading to better cosmetic results, faster recovery and high patient satisfaction.

## Case description

### Patient information

A 13-year-old girl was admitted on July 24, 2025 with left heel pain following a fall from height 3 h prior. X-ray revealed a comminuted fracture of the left calcaneus. She was subsequently transferred to the Department of Bone and Joint Rehabilitation for further management, where a diagnosis of left calcaneal fracture was confirmed.

### Clinical findings

**Figure 1:**
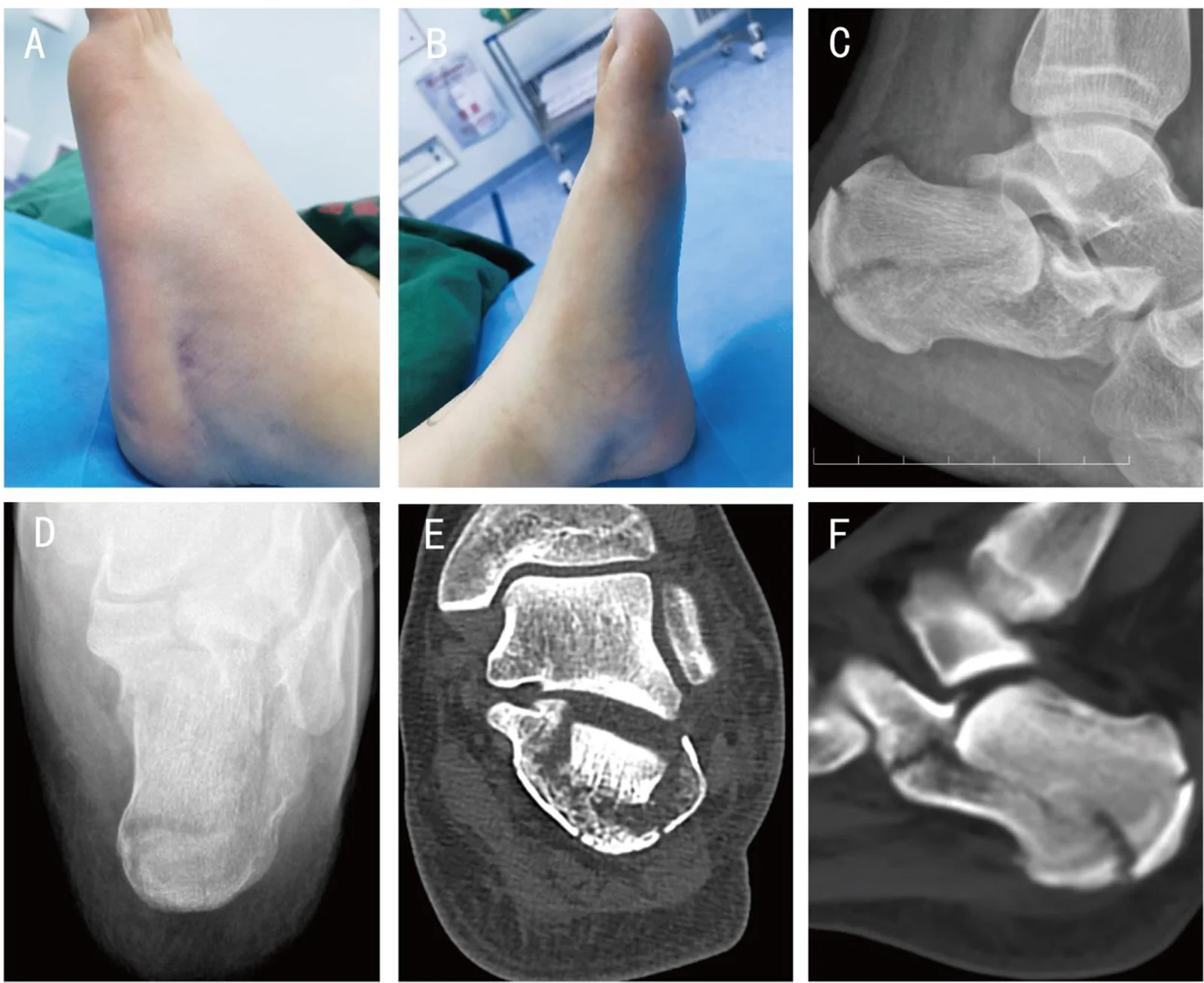
Preoperative assessment. **(A)** Lateral view of the left foot; **(B)** Medial view; **(C)** Lateral radiograph; **(D)** Axial radiograph; **(E)** Coronal CT; **(F)** Sagittal CT.

On examination, the left heel was swollen with ecchymosis but no open wound or tension blisters (Figures 1A,B). Tenderness was localized over the calcaneus. Toe movements and dorsalis pedis pulse were normal.

### Diagnostic assessment

Radiographs (Figures 1C,D) showed a comminuted calcaneal fracture with depression of the posterior facet and widening of the calcaneal body. Non-contrast CT with 3D reconstruction (Figures 1E–F) confirmed a Sanders type II fracture: a tongue-type fragment with posterior facet depression and lateral wall blowout. No other injuries were identified.

### Therapeutic intervention

**Figure 2:**
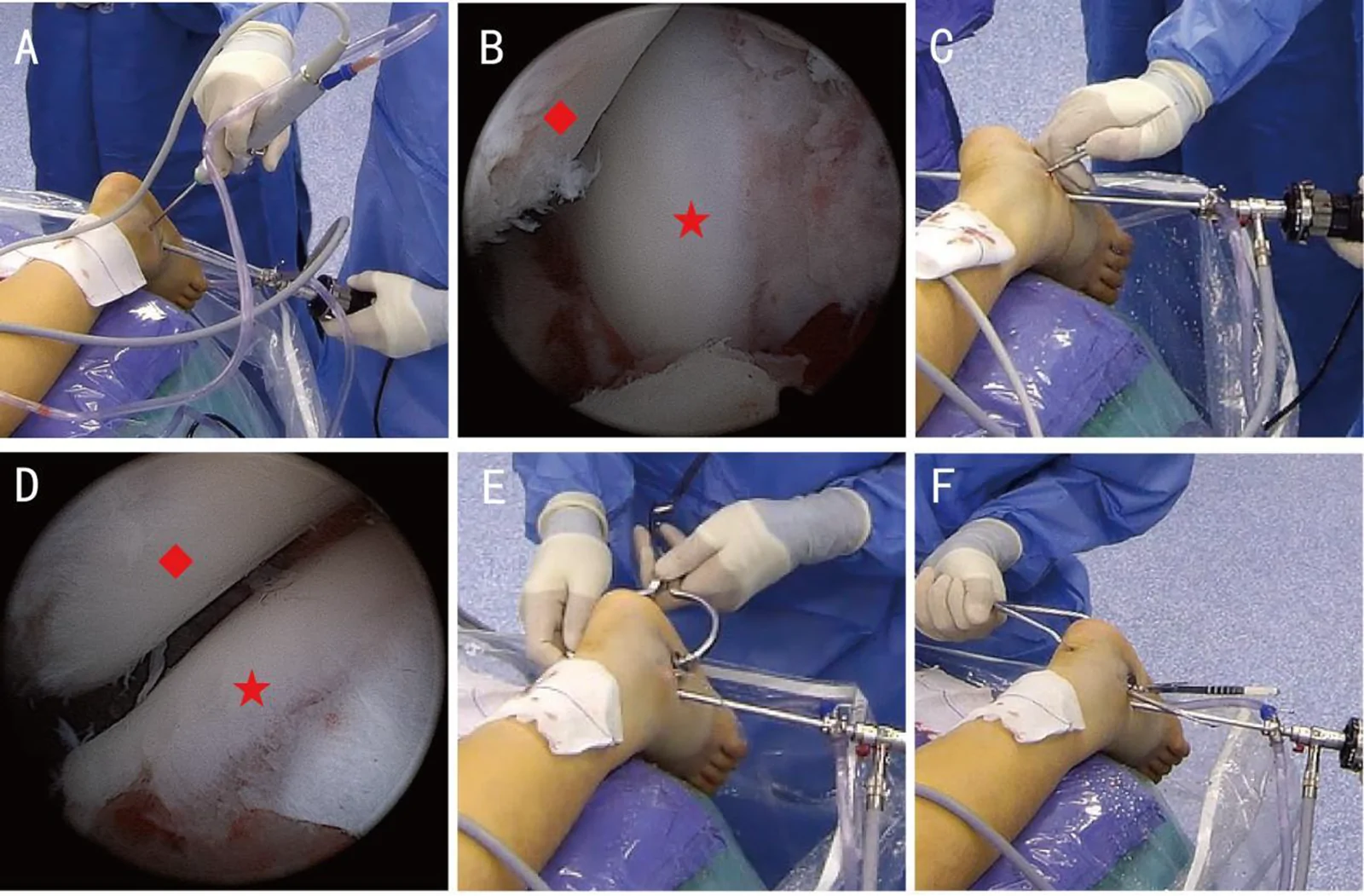
Intraoperative images and arthroscopic views. **(A)** Debridement via lateral portal (view from anterior portal); **(B)** Lateral portal arthroscopy: depressed posterior calcaneal facet (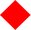, talus; 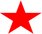, posterior calcaneal facet); **(C)** Elevation of depressed posterior facet fragment through lateral wall fracture gap; **(D)** Lateral portal arthroscopy after reduction: restored articular alignment (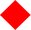, talus; 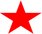, posterior calcaneal facet). **(E)** Percutaneous calcaneal reduction clamp compressing lateral wall. **(F)** Percutaneous pointed reduction clamp closing tongue-type fragment diastasis at calcaneal tuberosity.

Under general anesthesia, the patient was placed prone with a pneumatic tourniquet. Two 4-mm portals were created: an anterior portal, located 4 cm anterior to the lateral malleolus tip, for the arthroscope; and a lateral portal, located 1 cm inferior to the tip, for instruments. Subtalar arthroscopy revealed a depressed posterior facet fragment and a fracture line on the lateral wall (Figures 2A,B). A periosteal elevator was introduced through the lateral portal to elevate the fragment via the fracture gap, achieving anatomical reduction under direct vision (Figures 2C,D). A calcaneal reduction clamp was applied percutaneously to narrow the widened heel, and a pointed reduction clamp reduced the tongue-type diastasis (Figures 2E,F).

**Figure 3:**
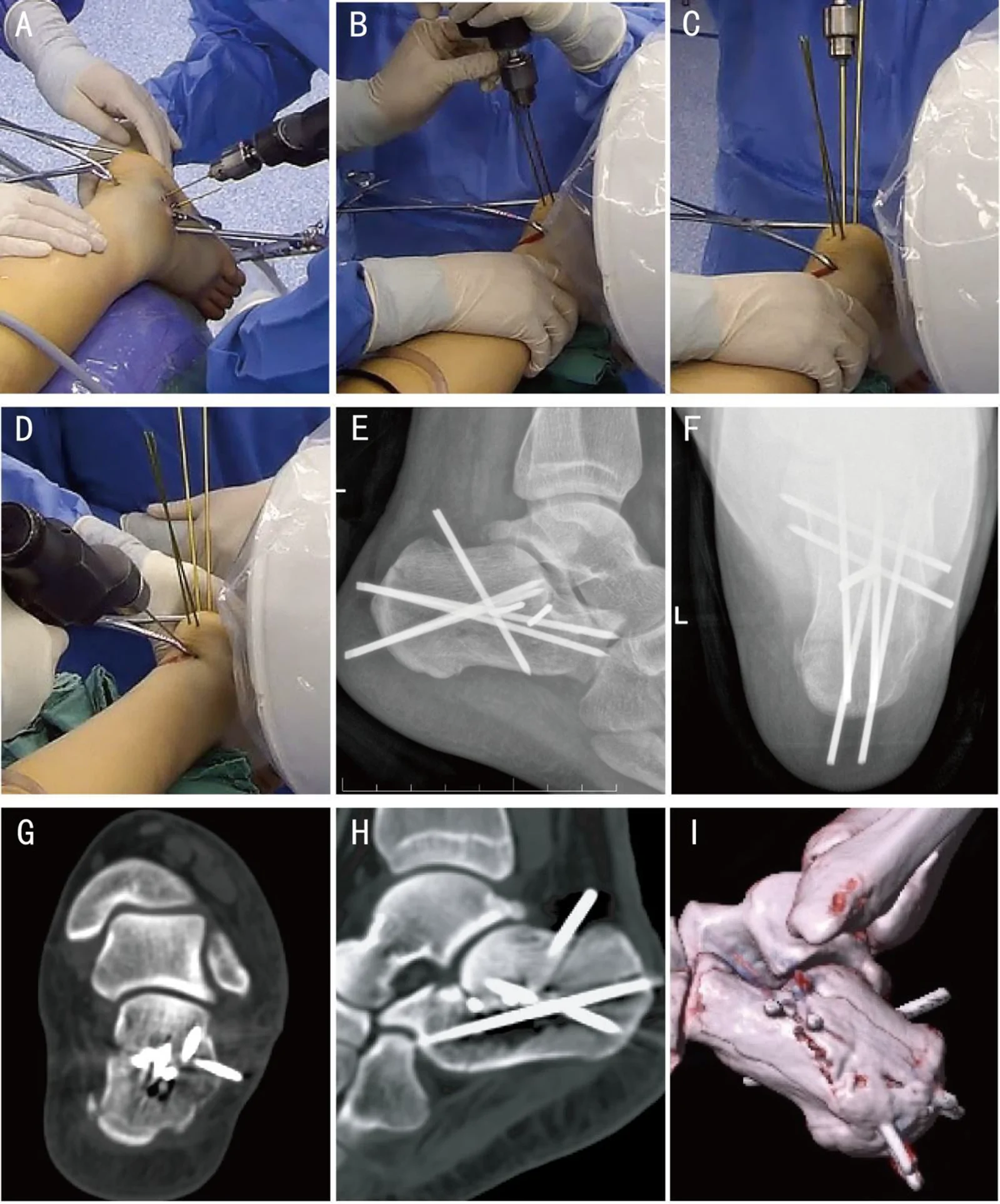
Intraoperative images of percutaneous Kirschner wire fixation: **(A)** Two 1.5-mm Kirschner wires inserted vertically below the posterior facet on the lateral wall; **(B)** Two 2.0-mm Kirschner wires driven from the posterior calcaneal tuberosity toward the calcaneocuboid joint; **(C)** Two 2.0-mm Kirschner wires placed from the plantar side of the tuberosity toward the posterior facet; **(D)** One 2.0-mm Kirschner wire inserted from the dorsal side of the tuberosity in an anteroinferior direction. Postoperative radiographs of the left calcaneus: **(E)** Lateral view; **(F)** Axial view. Postoperative CT images with 3D reconstruction of the left foot: **(G)** Coronal plane; **(H)** Sagittal plane; **(I)** 3D reconstruction.

The tourniquet was released, and percutaneous fixation was subsequently performed utilizing Kirschner wires as follows: first, two 1.5-mm Kirschner wires were inserted vertically from the lateral aspect, inferior to the posterior facet, to stabilize the reduced posterior facet fragment; second, two 2.0-mm Kirschner wires were driven from the posterior aspect of the calcaneal tuberosity toward the calcaneocuboid joint; third, two additional 2.0-mm Kirschner wires were placed from the plantar aspect of the tuberosity, directed posterosuperiorly towards the posterior facet; finally, one 2.0-mm Kirschner wire was inserted from the dorsal aspect of the tuberosity in an anteroinferior direction (Figures 3A–D). The protruding ends of the Kirschner wires were subsequently cut and countersunk. The minimally invasive portals were sutured, and a sterile dressing was applied. Postoperatively, the patient received a regimen of anti-inflammatory, decongestive, and thromboprophylactic therapy, in addition to early rehabilitation intervention. Follow-up lateral and axial radiographs (Figures 3E,F) and a non-contrast CT scan with three-dimensional reconstruction of the left foot (Figures 3G–I) indicated satisfactory reduction and fixation of the left calcaneal fracture.

The operative time for our minimally invasive procedure was 115 min, demonstrating favorable efficiency relative to the variable duration reported for traditional open reduction, which depends on surgeon proficiency and fracture severity.

### Follow-up and outcomes

**Figure 4:**
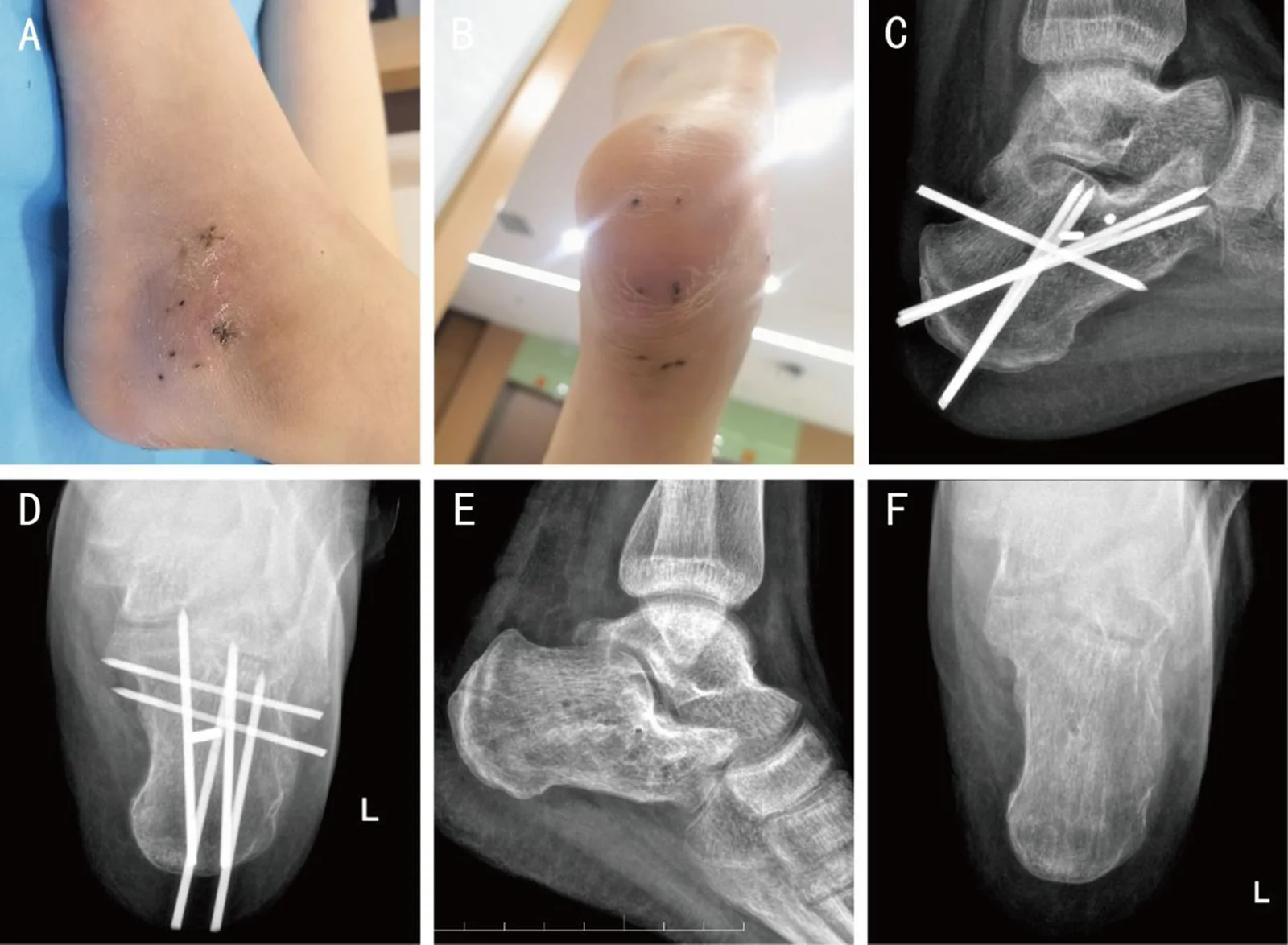
Postoperative follow-up. **(A)** Healed wound, lateral view; **(B)** Healed wound, posterior view; **(C)** Lateral radiograph before Kirschner wires removal; **(D)** Axial radiograph before Kirschner wires removal; **(E)** Lateral radiograph after Kirschner wires removal; **(F)** Axial radiograph after Kirschner wires removal.

Postoperatively, the patient experienced significant pain relief and reduced foot swelling, with well-preserved ankle and toe mobility and uneventful wound healing (Figures 4A,B). No postoperative complications were observed. Follow-up evaluations were conducted at 1, 2, and 3 months. At the 2-month follow-up, radiographs showed resolution of the fracture lines (Figures 4C–F). Considering the patient's intense fear of procedural pain, Kirschner wire removal was electively performed under general anesthesia via readmission. The patient resumed full weight-bearing ambulation. At the 3-month follow-up, the AOFAS Ankle-Hindfoot Scale score was 95 points (Table 1).

**Table 1:** Timeline of clinical treatment and postoperative follow-up.

Date	Event
Day 0	Injury, admission, imaging
Day 1	Surgery (arthroscopy-assisted reduction + Kirschner wires fixation)
Post-op day 1	Start active ankle/toe movements
1 month	Clinical follow-up: wounds healed, no pain
2 months	Radiographs show union; Kirschner wires removed; full weight-bearing initiated
3 months	AOFAS score 95; return to normal activities

## Discussion

Pediatric calcaneal fractures, though uncommon, are the most prevalent tarsal bone injury in children, with a notably higher incidence among those aged 11–15 years (3). These fractures typically result from high-energy trauma, most frequently falls from heights, where vertical compressive force affects the posterior facet of the subtalar joint, leading to calcaneal depression, heel widening and flattening, and a reduction in the tuber joint angle (4). Intra-articular fractures of this nature commonly involve articular impaction and bone loss (5). Given the calcaneus's role as the shared posterior pillar of the medial and lateral longitudinal arches, such injuries significantly impact foot function and frequently necessitate surgical intervention to restore Böhler's angle, Gissane's angle, the weight-bearing axis, and calcaneal width, while preserving or restoring its horizontal and axial length (6).

Conservative management of calcaneal fractures is associated with suboptimal outcomes and a high complication rate. Open reduction and internal fixation (ORIF) remains the predominant surgical technique; however, due to the complex anatomy of the calcaneus, traditional ORIF carries a consistently high rate of postoperative complications (7). Wound-related complication rates following the conventional extensile approach range from 2% to 27% (8). Conventional plates, locking plates, and various small-fragment sinus tarsi plates represent forms of eccentric fixation. Although these methods offer benefits such as extensive exposure for direct visualization and relative procedural simplicity, they are frequently accompanied by clinical challenges, including a notable prevalence of wound complications, eccentric plate–screw fixation is located lateral to the mechanical axis of the calcaneus, which generates substantial lever arm stress, resulting in inherent mechanical instability and an elevated risk of implant loosening, failure, screw breakage and screw pull-out (9).

Research on minimally invasive treatments for calcaneal fractures has focused on achieving reliable fixation without extensive open reduction. Techniques such as intramedullary nailing, internal fixation through a small sinus tarsi incision, and percutaneous reduction with Kirschner wire fixation are particularly suitable for patients with contraindications to open surgery, compromised soft tissue conditions, and Sanders type II–III fractures (10–13). Chu et al. (14) reported that both open reduction with plate fixation and closed reduction with percutaneous leverage and K-wire fixation yielded favorable outcomes for displaced intra-articular calcaneal fractures in older children. Although no significant difference in fracture healing time was observed between the two groups, open reduction resulted in superior postoperative recovery of foot and ankle function, attributed to the challenges of achieving direct visualization for posterior facet reduction during percutaneous leverage, which may lead to suboptimal articular surface alignment. With technological advancements, arthroscopic techniques have become increasingly prevalent in fracture surgery, enhancing the application of subtalar arthroscopy in treating calcaneal fractures. Subtalar arthroscopy facilitates thorough evaluation of articular cartilage damage and enables direct visualization of articular surface reduction (15). Currently, subtalar arthroscopy is recommended for Sanders type II–III fractures. For severe and complex fractures such as Sanders type IV, traditional open surgical methods remain essential. Indiscriminate application of minimally invasive techniques should be avoided, as inappropriate surgical decisions may adversely affect patient recovery (16–18).

In the present case, subtalar arthroscopy-assisted reduction and internal fixation with Kirschner wires was successfully performed. This technique offers several advantages over traditional open reduction and internal fixation. It eliminates the need to delay surgery until peri-fracture soft tissue stabilization or complete resolution of foot swelling, thereby reducing preoperative waiting time and hospitalization costs. Its minimally invasive nature decreases surgical trauma, improves cosmetic outcomes, and lowers the risk of severe complications such as infection, suppuration, and nonunion. These advantages are closely tied to the enhanced visualization and precision afforded by arthroscopy. The subtalar arthroscope provides direct visualization of the posterior calcaneal facet, enabling precise assessment of fracture reduction, while percutaneous leverage reduction through the lateral wall fracture gap facilitates anatomical restoration and minimizes reliance on intraoperative fluoroscopy. This combined approach also allows effective evaluation of peri-calcaneal joint stability and periarticular ligament integrity, reducing the likelihood of missing concomitant injuries.

Furthermore, the arthroscopic approach permits thorough debridement of fracture fragments and hematomas, alleviating postoperative joint swelling and lowering the risk of post-traumatic arthritis and intra-articular loose bodies. Intraoperative blood loss is minimized, and the tourniquet can be released immediately after reduction, shortening tourniquet time and reducing associated complications. Preservation of periosteal integrity promotes bone healing, reduces postoperative pain, and improves foot and ankle function. From a biomechanical perspective, intramedullary crossing fixation with Kirschner wires provides stable support for both the articular surface and the mechanical axis of the calcaneus. The buried Kirschner wire ends reduce irritation to overlying soft tissues, and the implants can be removed through the same minimally invasive incisions after fracture union.

Despite these advantages, certain limitations should be acknowledged. This technique requires proficiency in subtalar arthroscopy and a thorough understanding of calcaneal anatomy, presenting a steep learning curve. In addition, severely comminuted fractures, particularly Sanders type IV, may not be suitable for arthroscopic reduction due to the lack of dedicated instruments and the need for extensive reconstruction. Nevertheless, continued technological advancements and instrument innovation are expected to broaden the applicability of this approach.

## Data Availability

The original contributions presented in the study are included in the article/Supplementary Material, further inquiries can be directed to the corresponding author.
