# Correction: Case Report: Arthroscopic minimally invasive treatment of pediatric calcaneal fracture

**DOI:** 10.3389/fped.2026.1982368

**Published:** 2026-09-09

**Authors:** Li Zhiye, Wang Ye, Ren Zhaohui, Lv Guangren

**Affiliations:** Bone and Joint Rehabilitation Center, Xiangya Boai Rehabilitation Hospital, Changsha, Hunan, China

**Keywords:** internal fixation, minimally invasive surgery, pediatric calcaneal fracture, percutaneous leverage reduction, subtalar arthroscopy

There was a typo error regarding the patient admission year in the Case description section.

A correction has been made to the section Case description, subsection Patient information, Paragraph 1: “The original sentence: A 13-year-old girl was admitted on July 24, 2020 with left heel pain following a fall from height 3 h prior.” The corrected sentence: “A 13-year-old girl was admitted on July 24, 2025 with left heel pain following a fall from height 3 h prior.”

The original version of this article has been updated.

